# The Efficacy and Safety of Oral Irrigator on the Control of Dental Plaque and Gingivitis: A Randomized, Single-Blind, Parallel-Group Clinical Trial

**DOI:** 10.3390/ijerph20043726

**Published:** 2023-02-20

**Authors:** Xiaolin Ren, Jing He, Ran Cheng, Yulun Chen, Yong Xiang, Yuhan Zhang, Sulan Jiang, Jia Li, Li Cheng, Tao Hu

**Affiliations:** 1 State Key Laboratory of Oral Diseases, National Clinical Research Center for Oral Diseases, Department of Preventive Dentistry, West China Hospital of Stomatology, Sichuan University, Chengdu 610041, China; 2 State Institute of Drug Clinical Trial, West China Hospital of Stomatology, Sichuan University, Chengdu 610041, China

**Keywords:** oral hygiene, oral irrigator, dental plaque, gingivitis, clinical trial

## Abstract

Background: To evaluate the efficacy and safety of oral irrigator (OI) in controlling dental plaque and gingivitis. Methods: Ninety participants diagnosed with gingivitis were randomly assigned to two groups, given a toothbrush combined with OI (WaterPik^®^) (test) or a toothbrush alone (control). The Turesky-Modified Quigley-Hein Plaque Index (T-QH), Modified Gingival Index (MGI), Bleeding Index (BI), and percentage of sites with bleeding on probing (BOP%) were evaluated at baseline, 4 weeks, 8 weeks, and 12 weeks. The full analysis set (FAS) and per-protocol set (PPS) were analyzed. Adverse events were recorded through electronic diaries and examinations. Results: Of the 90 participants, the efficacy was assessed in the following numbers (FAS/PPS): test (45/33) and control (43/38). Compared with the control, MGI, BI, and BOP% were significantly lower in the test group after 4 weeks (4 weeks: *p* = 0.017, *p* = 0.001, and *p* = 0.001, respectively; 8 weeks and 12 weeks: *p* < 0.001 for all, FAS); T-QH was significantly lower after 8 weeks (8 weeks: *p* = 0.033; 12 weeks: *p* = 0.006, FAS). Transient gingival bleeding may be associated with OI. Self-reported pain and dentin hypersensitivity symptoms were similar between groups. Conclusions: As adjuncts to toothbrushing, OI demonstrated significantly better efficacy in controlling dental plaque and gingival inflammation with no substantial safety hazards.

## 1. Introduction

Periodontal disease is a common disease that may bring severe implications for oral and systemic health and negatively impact the patient’s quality of life [1]. According to the fourth national oral health survey in mainland China, almost 90% of Chinese adults suffer from periodontal disease of various severities [2]. Plaque biofilm is considered the initiating factor of periodontal disease [3]. In the presence of other risk factors (e.g., individual susceptibility), the symbiotic balance between the host and microbiota may be lost, resulting in disease development [4]. In daily practice, patients’ self-performed biofilm removal might be limited. Interproximal surfaces and sites with special anatomic and morphologic conditions may not be adequately cleaned with conventional toothbrushing [5,6]. Many interdental cleaning devices have been widely investigated as adjuncts to toothbrushing [7,8]. Different interdental toothbrush shapes and sizes should be considered to accommodate different interdental spaces, and they may not apply to anterior teeth with small and insufficient interdental space. Consequently, flossing may be an alternative for healthy sites without attachment loss to avoid potential trauma [9]. However, according to a national epidemiological study, only 2.6% of Chinese adults floss daily [10]. In addition, flossing requires a high level of hand dexterity and good anatomic knowledge, which may reduce self-performed flossing effectiveness [6]. The demand for more time-efficient and convenient interdental cleaning devices is growing.

Compared with manual floss, oral irrigators (OIs) could be potential interdental cleaning devices. OIs, introduced to the public in the 1960s, are generally designed to remove soft debris and unattached plaque through the mechanical action of a pulsating stream of water [11]. The available evidence for OIs to relieve plaque and gingivitis is limited and inconsistent [12]. Several systematic reviews showed that OIs failed to reduce visible plaque as an adjunct to toothbrushing but improved gingival inflammation-related indices [12,13]. Evidence also supports that supragingival irrigation may benefit people with poor oral hygiene or lack of interproximal cleaning [11,13,14]. Besides, OIs in early studies had a fixed or limited range of pressure settings, generally 50~60 pound per square inch (Psi) [15,16,17], 70 Psi [18], or 80 Psi [19,20], etc. Furthermore, the present studies focus on the short-term (about four weeks) effect and lack longer-term assessments [21,22,23]. To fulfil the needs of different groups of people, water pressure of the current OIs could change from 10 to 100 Psi. The longer-term effect of OIs with a wide range of pressures has yet to be fully studied.

Except for efficacy, less attention has been given to the safety of OIs, especially those with maximum pressures of 100 Psi. Oral tissue assessments and participants’ diaries are the primary methods to assess the safety of OIs [12]. In healthy people, OIs generally cause no or only minor adverse events, such as oral lacerations [24]. However, OIs could generate water jets with high fluid shear stress and high impact pressure to detach dental biofilms [25]. It remains unclear whether the irrigation forces cause pain and dentin hypersensitivity symptoms. Thus, self-reported pain and dentin hypersensitivity symptoms with electronic visual analogue scales (VAS) were assessed in this trial.

This study aimed to investigate the efficacy and safety of OIs with broader and adjustable pressure settings in managing dental plaque and gingival inflammation for patients with gingivitis during 12 weeks of follow-up. It is hypothesized that, compared to manual toothbrushing only, this OI as an adjunct would improve plaque and gingival inflammation-related indices in patients with gingivitis without causing serious adverse events and pain and dentin hypersensitivity symptoms.

## 2. Materials and Methods

### 2.1. Study Design and Ethical Considerations

A 12-week randomized, single-blind, parallel-group clinical trial was conducted at West China Hospital of Stomatology, Sichuan University, Chengdu, China. The research program was approved by the Research Ethics Committee of West China Hospital of Stomatology, Sichuan University (WCHSIRB-D-2021-493). This study was conducted in full compliance with the Declaration of Helsinki, the related regulations of the People’s Republic of China, and Good Clinical Practice. This trial was retrospectively registered in the Chinese Clinical Trial Registry (ChiCTR, Registration number: (ChiCTR2100054254)).

### 2.2. Sample Size

The sample size was calculated using G*power 3.1.9.7 based on the mean Modified Gingival Index (MGI) of 1.18 with a standard deviation of 0.2 in the OIs group according to a previous study data [26]. In total, 76 patients were required to estimate a 10% difference between the control and test groups, with a significance level of 5% and a power of 80%. In addition, considering the potential 20% attrition during follow-up, 90 participants were recruited for this study.

### 2.3. Eligible Criteria

#### 2.3.1. Inclusion Criteria

- Male and female aged 18 to 65 years old.
- In good general health with brushing teeth as a daily habit.
- Possessing at least 20 permanent teeth (excluding the third molars) and 5 evaluable teeth in each quadrant.
- Suffering from gingivitis and possessing at least 20 gingival bleeding on probing at the baseline examination.
- T-QH > 1.5 at baseline examination.

#### 2.3.2. Exclusion Criteria

- Pregnant or breastfeeding women.
- Cigarette smoking.
- Dental students, employees of the clinical research center, or product-related companies.
- Suffering from periodontitis or gingival recession.
- Using oral hygiene adjunct tools regularly (i.e., electric toothbrushes, dental floss, interdental brush, mouthwashes, etc.).
- Wearing orthodontic bands, orthodontic appliances, removable dentures, or gross prosthesis.
- Suffering from untreated dental caries, mucosal lesions, xerostomia, oral tumors, or severe systemic diseases that may affect periodontal diseases.
- Received professional periodontal scaling and root planning within four weeks, periodontal surgery within six months, or received oral and maxillofacial surgery within three months.
- Participated in another clinical trial within three months.
- Antibiotics, nonsteroidal anti-inflammatory drugs, Chinese medicine, or anticoagulant drugs are being taken.
- Allergies to the material used in the study product.

### 2.4. Randomization and Allocation

After enrollment based on the eligibility criteria, the central randomization method was applied based on computer software. The enrollment and randomization process were conducted by a third party that was unrelated to the clinical examination. The participants were arranged into control or test groups in a ratio of 1:1, with 45 participants in each group. Investigators were blinded to randomization and allocation concealment until the completion of the studies.

### 2.5. Interventions and Outcomes Measurement

After preliminary screening of personal medical history and oral examination at baseline, the participants were randomly assigned to one of the intervention groups: the toothbrush + oral irrigator group (test group) received an OI (WaterPik^®^ ION Professional Cordless Water Flosser, marketed as GT17 on this region, WaterPik, Inc., Fort Collins, CO, USA) with a uniform standard manual toothbrush (Crest^®^ Multicolored Crystal Soft Bristles, Procter and Gamble Co., Cincinnati, OH, USA) and a toothpaste (Crest^®^ Anti-Cavity and Enamel Repair Toothpaste, Procter and Gamble Co., Cincinnati, OH, USA); the toothbrush group (control group) received an identical toothbrush and toothpaste. The OIs allowed for 10 adjustable water pressure settings ranging from 10 to 100 psi, which corresponded to 10 different switches. At baseline, the participants in the test group were instructed to use the OIs in the clinic for the first time by an experienced dental assistant. Furthermore, they were instructed to use the standard jet tip to flush the gingival margin and interdental space twice a day for approximately 90 s before toothbrushing. According to the manufacturer’s recommendations, the participants were advised to adopt the most comfortable water pressure level with a minimum of four. In addition, all participants were instructed to perform manual toothbrushing twice a day with the modified Bass technique. Participants were distributed electronic diaries to record the frequency of toothbrushing and oral irrigation and any adverse reactions or discomfort symptoms. Compliance was evaluated based on the diaries, and it was defined as toothbrushing or oral irrigation ≥ 2 × daily in >80% of study days. Instruction leaflets with toothbrushing methods and oral irrigation instructions were distributed to participants, and they were advised not to use other oral hygiene adjuncts, such as dental floss, interdental brush, and gum.

Before every clinical examination, all participants were instructed to refrain from oral hygiene for 12 h and fast for solids and liquids for 2 h. A single trained examiner evaluated all gingival inflammation-related indices. The MGI [27] were examined at four sites (mesial buccal, buccal, distal buccal, and lingual) per tooth. The Bleeding Index (BI) [28] and percentage of sites with bleeding on probing (BOP%) were examined at six sites (mesial buccal, buccal, distal buccal, mesial lingual, lingual, and distal lingual) per tooth using community periodontal index (CPI) probes. Another trained examiner evaluated the Turesky-Modified Quigley-Hein Plaque Index (T-QH) [29] after applying the dental plaque disclosing agent. Furthermore, a series of safety observation indicators were evaluated. Gingival recession is defined as apical migration of the gingival margin, and clinically visible sites of gingival recession were examined and recorded. The VAS was adopted to assess the pain and dentin hypersensitivity symptoms during the trial [30]. The left end of the line segment is marked for no pain or sensitivity symptoms, while the right end represents the most severe pain or sensitivity symptoms. The participants were instructed to mark the line segment according to their existing symptoms during the last month. In addition, vital signs of allergic reactions or lesions in the soft tissue of the oral cavity, including the gingiva, buccal mucosa, lips, palate, vestibular sulcus, tongue, and floor of the mouth, were recorded.

Participants returned to the clinical research center for dental examination at 4 weeks ± 3 days, 8 weeks ± 3 days, and 12 weeks ± 3 days after baseline examinations. The T-QH and gingival inflammation-related indices, including MGI, BI, and BOP%, were reevaluated. Furthermore, the safety indicators were also recorded. The modified Bass technique and the method of OIs use were reinforced at each visit by the same dental assistant. The primary outcomes were gingivitis-related indices after 12 weeks of using OIs. The secondary outcomes were plaque-related indices and gingivitis-related indices at 4 weeks and the safety indicators.

### 2.6. Statistical Analysis

The efficacy evaluation was analyzed with full analysis set (FAS) based on the intention to treat principle and the per-protocol set (PPS), and the safety evaluation was based on the safety set (SS). The FAS comprised participants who had received at least one post-intervention assessment after randomization. The PPS included participants who completed the trial without significant protocol deviations that could have affected the outcomes. The last observation carried forward (LOCF) was adopted for imputing the missing values of results. Analyses were performed by an independent company (Vantage Marketing and Research Consultants Ltd., Guangzhou, China) using statistical software (SPSS^®^, Version 25, Chicago, IL, USA). The normal distribution for indices’ values was examined by the Shapiro–Wilk test. ANOVA or independent samples *t* tests were adopted for normally distributed variables, and Wilcoxon rank-sum tests were adopted for non-normally distributed variables for intergroup comparison. Chi-squared tests or Fisher’s exact tests were used to compare distribution frequency. Two-way repeated measures ANOVA and post hoc tests with Bonferroni correction were performed to compare intragroup differences between time points. Friedman’s tests were instead for ANOVA for non-normally distributed repeated measured variables. The association between variables was measured with the Pearson correlation or Spearman rank correlation depending on data characteristics. All tests were two-sided, and significant threshold of *p* < 0.05 was adopted.

## 3. Results

**Figure 1:**
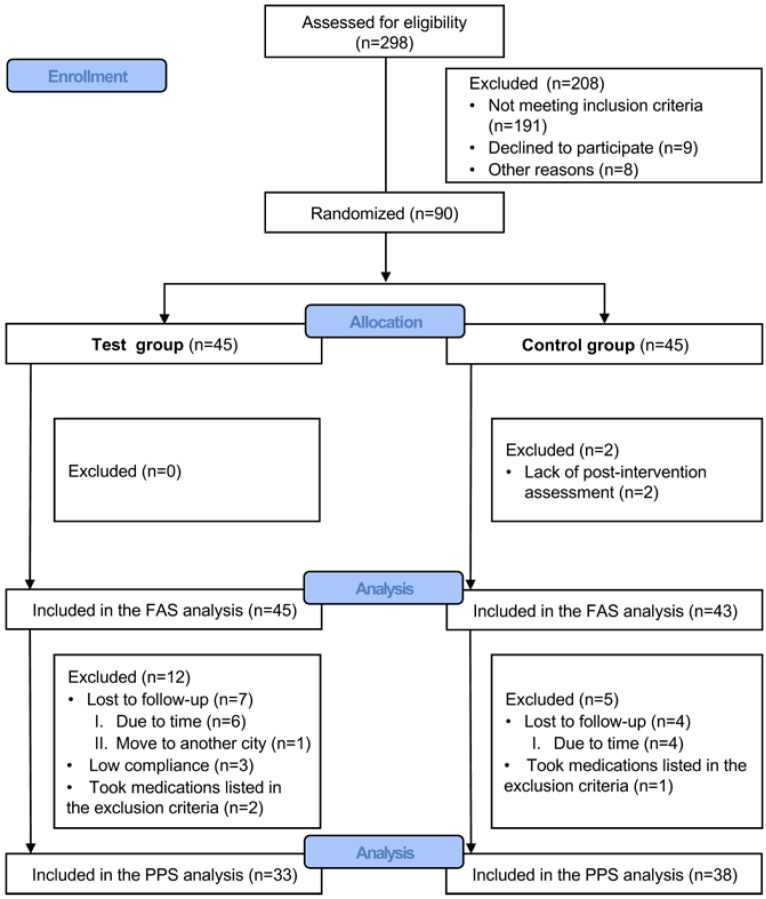
CONSORT flow diagram of the trial. FAS, full analysis set; PPS, per-protocol set.

In total, 298 participants were screened; 90 participants were included in this trial, and they were equally allocated into the test and control groups. Two participants in the control group dropped out of the study after baseline. Therefore, they were not included in the FAS due to lacking post-intervention data. Overall, 88 participants were included in the FAS analysis. Among them, 71 (80.7%) participants, including 33 participants in the test group and 38 in the control group, were included in the PPS analysis. The most common reason for exclusion from PPS analysis was lost to follow-up (n = 13). One participant in the test group dropped out at the 8 weeks visit. Furthermore, six participants in the test group and four in the control group were lost to follow-up at the 12 weeks visit. In addition, three participants in the test group were excluded from PPS due to low compliance (<80%). Two participants in the test group and one in the control group were also excluded from PPS because they took medications listed in the exclusion criteria. The flow diagram is exhibited in Figure 1.

The periodontal indices and the demographic data, including gender, ethnicity, age, and education level, between the two groups were well-balanced at baseline, which reflected that the process of randomization was effective. The mean age between groups was similar (30.67 ± 10.05 in the test group, 30.49 ± 11.64 in the control group, *p* = 0.314), with more females than males. There was no statistically significant difference in the baseline plaque and gingival inflammation-related indices (*p* > 0.05 for all). Table 1 provides details of the demographic characteristics and the periodontal indices at baseline. Calculating “unclean” as irrigation pressure to zero, the average pressures participants used during the trial ranged from 38.554 to 82.353 Psi (52.133 ± 11.04) according to the electronic diary. Average irrigation pressures were positively correlated with the reduction in percentage of BOP% at the baseline–12 weeks period (rho = 0.330, *p* = 0.027, Figure S1), while there was no significant correlation with the reduction in percentage of T-QH, MGI, and BI (*p* > 0.05 for all, Figure S1). Similar results were found for the PPS (Figure S2).

The comparison between the two groups during the follow-up period in the FAS is shown in Table 2. Gingival inflammation-related indices (MGI, BI, and BOP%) were significantly improved compared with the control group at the 4 weeks visit (*p* = 0.017, *p* = 0.001, *p* = 0.001, respectively, FAS), while T-QH scores were not significantly improved (*p* = 0.102, FAS). In the PPS (Table S1), the BI and BOP% in the test group were also improved at 4 weeks visit (*p* = 0.014, *p* = 0.020, respectively), although the MGI scores were not significantly different *(p* = 0.082). All indices (T-QH, MGI, BI, BOP%) were significantly lower compared with those of the control group at the 8 weeks and 12 weeks visits (T-QH: *p* = 0.033, 8 weeks; *p* = 0.006, 12 weeks; *p* < 0.001 for others, FAS). The PPS analysis shows similar results.

**Figure 2:**
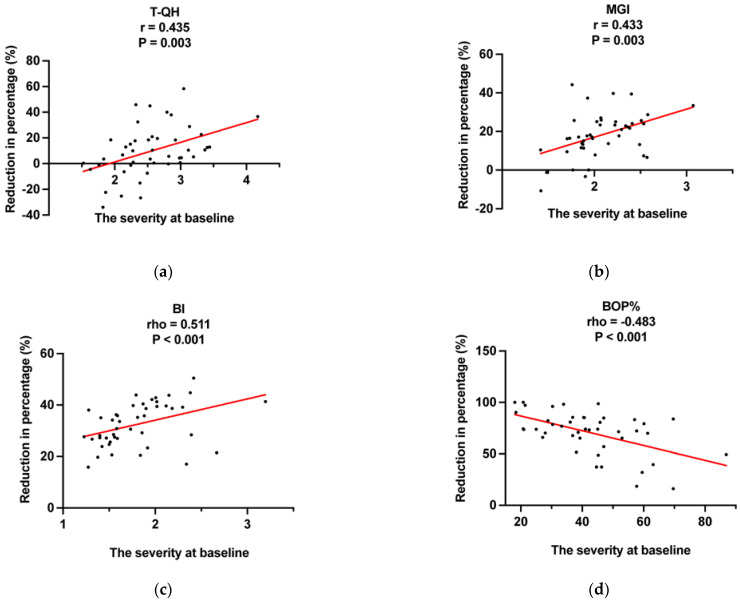
The correlation between the reduction in percentage at the baseline–12 weeks period and the severity at baseline (full analysis set). (**a**) T-QH, Turesky-Modified Quigley-Hein Plaque Index; (**b**) MGI, Modified Gingival Index; (**c**) BI, Bleeding Index; (**d**) BOP%, percentage of sites with bleeding on probing. Pearson correlation analysis was adopted in (**a**,**b**). Spearman correlation analysis was adopted in (**c**,**d**). r, Pearson correlation coefficient; rho, Spearman correlation coefficient.

Table 3 compares the reduction in percentage in T-QH, MGI, BI, and BOP% between groups at different time points in FAS. Table S2 shows the results for PPS. At the 12 weeks–baseline period, all indices (T-QH, MGI, BI, BOP%) in the test group showed more significant decreases in both FAS and PPS. In the FAS, significantly higher reductions were found for BI and BOP% in the test group at the baseline–4 weeks period (*p* < 0.001, *p* < 0.001, respectively). Moreover, significantly higher reductions were found for MGI and BOP% in the test group at the 4 weeks–8 weeks period (*p* = 0.007, *p* < 0.001, respectively). Nevertheless, the PPS analysis revealed significantly higher reduction for all gingival inflammation indices at the baseline–4 weeks and 4 weeks–8 weeks period. The reduction in percentage of all indices in FAS and PPS were similar between groups in the 8 weeks–12 weeks interval (*p* > 0.05 for all). As shown in Figure 2, the reduction in percentage in T-QH, MGI, and BI at the baseline–12 weeks intervals were positively correlated with baseline scores (r = 0.435, *p* = 0.003; r = 0.433, *p* = 0.003; rho = 0.511, *p* < 0.001, respectively), while the reduction in percentage in BOP% was negatively correlated with baseline scores (rho = −0.483, *p* < 0.001). The PPS analysis demonstrates similar results (Figure S3).

**Figure 3:**
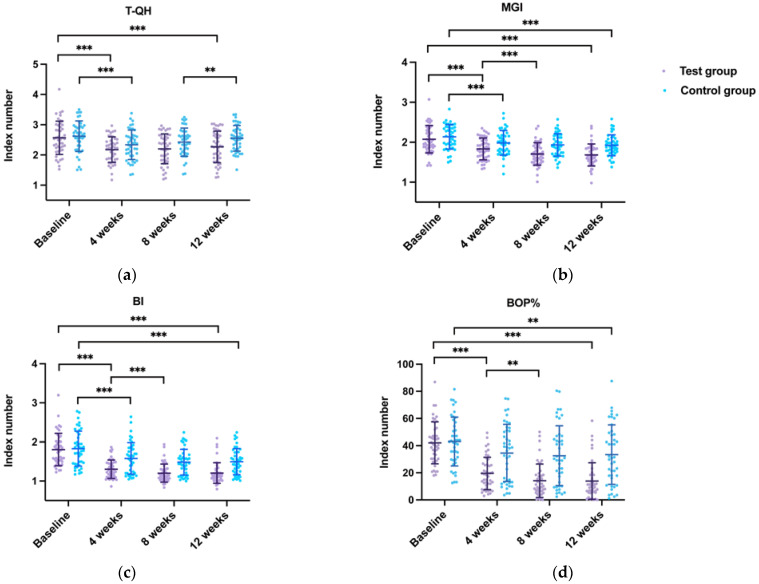
Scatter diagram of periodontal indices for two groups at follow-up (full analysis set). (**a**) T-QH, Turesky-Modified Quigley-Hein Plaque Index; (**b**) MGI, Modified Gingival Index; (**c**) BI, Bleeding Index; (**d**) BOP%, percentage of sites with bleeding on probing. Intragroup comparisons in (**a**,**b**) were performed using two-way repeated measures ANOVA and post hoc tests with Bonferroni correction. Intragroup comparisons in (**c**,**d**) were performed using Friedman’s test (** *p* < 0.01, *** *p* < 0.001).

Furthermore, the comparison between the baseline–12 weeks period and adjacent visit points (baseline–4 weeks, 4 weeks–8 weeks, and 8 weeks–12 weeks periods) in FAS are present in Figure 3. During the baseline–12 weeks period, all gingival inflammation-related indices significantly decreased in both groups (*p* < 0.001 for all, except BOP% in the control group, *p* = 0.004, FAS); the T-QH in the test group significantly decreased (*p* < 0.001, FAS), while in the control group there were insignificant changes (*p* = 1.000, FAS). T-QH, MGI, and BI in both groups and BOP% in the test group decreased significantly during the baseline–4 weeks period (*p* < 0.001 for all, FAS). During the 4 weeks–8 weeks period, the MGI, BI, and BOP% decreased significantly in the test group (*p* < 0.001, *p* < 0.001, and *p* = 0.008, respectively, FAS), while no significant difference was found in the control group (*p =* 0.321, *p* = 0.244, and *p* = 1.000, respectively, FAS). The T-QH scores in the control group increased significantly for the 8 weeks–12 weeks period (*p* = 0.004, FAS) and tended to increase in the test group, but the difference was not statistically significant (*p* = 0.395, FAS). Similar results are found in PPS (Figure S4).

A comprehensive assessment of adverse events in the safety set was carried out through electronic diaries and clinical examinations. Overall, 37 adverse events were found during the trial, and none were severe adverse events. Among them, Transient gingival bleeding may be associated with the OIs. Three participants in the test group reported this adverse event, with an incidence of 6.67% (3/45). Moreover, a similar number of adverse events were observed between the two groups (test: 18/45 (40.0%); control: 19/43 (44.2%), *p* = 0.829, Table 4).

In terms of pain and dentin hypersensitivity symptoms (Table 5), most participants experienced pain and dentin hypersensitivity levels close to 0 (Q_2_ = 0, in both groups at all time points). Additionally, no significant difference in VAS value was identified between two groups during 12 weeks of follow-up (*p* > 0.05 for all). There were no clinically examinable changes in gingival recession in the two groups during the trial. No OIs-related soft or hard tissue trauma was observed during the trial. Compliance was evaluated based on participants’ diaries. The median number of days the OI was used was 98.81%, with the 25th percentile of 96.45%. Counting the missing values as “unclean”, the compliance with toothbrushing was close in both groups (*p* = 0.355, Table 6).

## 4. Discussion

This study evaluated the efficacy and safety of oral irrigators with broader range of pressures in removing plaque and relieving gingivitis. The test group showed significant improvement in plaque and gingivitis indices than the control group. Higher irrigation pressures significantly correlated with the reduction in percentage of BOP%. The OI may be associated with transient gingival bleeding without causing significantly higher of pain or dentin hypersensitivity symptoms. Both intention-to-treat-based and per-protocol analyses were performed for efficacy evaluation to obtain more accurate results. The per-protocol analysis tends to exaggerate the treatment effect, and the results obtained from the intention-to-treat-based analysis more closely represent clinical practice and actual effectiveness [31]. Therefore, the results of the FAS were given precedence when conflicting results arose.

At baseline, all clinical parameters were similar in both groups. After 12 weeks, dental plaque and gingival inflammation were significantly improved in the test group compared with the control group in FAS and PPS, indicating that OIs effectively controlled plaque and relieved gingival inflammation. Significantly lower gingival inflammation indices in the test group were also observed at 4 weeks and 8 weeks. Compared to manual toothbrushing alone, the OIs group was significantly more effective in controlling gingival inflammation, which is consistent with the findings of most studies [13,19,32,33,34]. Nevertheless, inconsistent evidence exists for the efficacy of the OIs in reducing dental plaque, suggesting a significant improvement in the OIs group [21,33,34] or no significant difference between groups [12,13,14,19]. In the current study, despite the difference in T-QH between the two groups was not significant at 4 weeks, there was a significant improvement in the test group at 8 and 12 weeks. These results are similar to the previous finding, which also found no significant change in plaque index in the short term (6 weeks) but a significant improvement in the later period (12 weeks) [33]. The efficacy of OIs for plaque cleaning at different time points may require further research.

The reduction in percentage of all gingival inflammation-related indices and plaque index in the test group were also significantly higher compared with control group in FAS and PPS. During the 12 weeks follow-up, a decreasing trend of gingival inflammation-related indices was observed in both groups. However, plaque in both groups showed an upward trend after the 4 weeks visit, with gingival inflammation remaining relatively low, which may be explained by the previous study’s findings [35]. The increase in plaque may not lead to a correlated increase in gingival inflammation for patients performing daily oral hygiene measures when plaque formation/maturation was effectively interrupted daily [35].

As shown in Figure 2 and Figure S3, positive correlations were found between baseline T-QH and MGI scores and the reduction in percentage. The BI was a 0–5 scale index, with 0 being the normal-appearing, healthy gingiva and 5 being spontaneous bleeding. It was positively correlated with the reduction in percentage, while baseline BOP% was negatively correlated. In combination, OIs may reduce bleeding severity more than the number of bleeding sites. In addition, the plaque- and gingival inflammation-related indices in the control group also showed significant improvement. This may be related to the modified Bass brushing method. On the other hand, the Hawthorne effect may influence the observed effects, especially as participants were not blinded and knew they were being observed [36].

The irrigation pressure seems to be positively correlated with the efficacy of OIs in relieving gingival inflammation. However, the traumatic injuries that may be associated with high-pressure water flow generated by the OIs have also gained attention [37]. Safe irrigation pressure should consider the patient’s periodontal status. Previous studies have yielded different pressure ranges, such as 90 psi for undamaged gingival tissue, 50–70 psi for inflamed or ulcerated oral tissue [38], 83–87 psi for moderate to advanced periodontitis patients [39], and 60 psi for periodontal pockets [40]. In this study, the participants were required to adopt the most comfortable irrigation pressure with an average range from 38.554 to 82.353 Psi, which is considered safe and tolerable for gingivitis patients. This is in line with the American Academy of Periodontology position paper, where irrigation pressure of 80–90 psi was considered tolerable [11]. In addition, the safety of OIs at maximum pressure may need further study.

Regarding safety, previous research has investigated a variety of systemic and soft tissue-related indicators and showed that OIs are generally not deleterious to people in good general health [11]. However, to the best of our knowledge, the relationship between pain or dentin hypersensitivity symptoms and OIs usage has not been investigated. In this trial, the OIs did not cause additional pain or dentin hypersensitivity symptoms compared to the control group. Gingival recession, associated with dentin hypersensitivity [41], also showed clinically examinable difference during follow-up. However, the results need to be interpreted with caution, as it takes time for gingival recession to appear as a clinically examinable sign. In longer-term six-month studies, the usage of OIs during the periodontal maintenance period also did not cause gingival recession [19,42].

Similar adverse events were found in the two groups. Despite the fact that transient gingival bleeding was found to be likely associated with OIs, gingival bleeding is considered one of the common symptoms of gingivitis [43]. In addition, patient compliance plays a vital role in oral hygiene practices. True compliance is commonly inconsistent with the high compliance recorded by a paper diary [44]. For this reason, an electronic diary was adopted in this trial due to its relatively high reliability [45]. The data showed that, although the majority of participants had good compliance with the OIs (25th percentile was 96.45%), three participants demonstrated low compliance (80%), and they were excluded from the PPS.

Oral irrigation appears to be an effective adjunct in managing gingivitis. Several mechanisms may be involved. For instance, OIs may remove food deposits and interfere with plaque maturation by flushing loosely adhered plaques [13]. Water irrigation may bring changes in plaque from a more subtle perspective and not be easily detectable with a two-dimensional scoring system, such as reducing the plaque’s toxicity or “thickness” [17]. In vitro studies also show that the mechanical force of the high-velocity water micro-sprays generated by OIs promotes the penetration of antimicrobial drugs into deeper biofilms and significantly reduces biofilm thickness [46]. In addition, oral irrigation may bring changes in subgingival microbiota [47]. The level of putative periodontal pathogens decreased after 6 weeks of subgingival irrigation with water [48]. Pro-inflammatory mediators and biomarkers may also change. Oral irrigation as an adjunct resulted in significantly reduced reactive oxygen species generation in blood samples from periodontitis patients. Furthermore, interleukin 1 beta (IL-1β) and Prostaglandin E2 (PGE2) were significantly reduced compared to the baseline [33]. Similarly, IL-1β and PGE2 levels were significantly decreased in the gingival crevicular fluid in the oral irrigation group compared to routine oral hygiene, while IL-10 levels were significantly increased [32]. Conflicting results also exist. A study revealed that matrix-metalloproteinase 3 (MMP-3), MMP-8, and IL-1β in gingival crevicular fluid and whole saliva of gingivitis patients remained insignificantly altered over 8 weeks of OIs use [49]. The mechanisms by which OIs control gingival inflammation and plaque may be multifaceted, requiring further research.

In this study, we evaluated the efficacy of the OIs in gingivitis patients based on both intention-to-treat and per-protocol analysis. We also analyzed the relationship between its efficacy and irrigation pressure. In terms of safety, we analyzed the relationship between OIs and self-reported symptoms of pain and dentin hypersensitivity using the VAS scale for the first time. However, there are still limitations in this study. For instance, we did not use other interdental cleaning tools (e.g., flossing and interdental brushes) for comparison with the efficacy of OIs. The superiority trials between OIs and other interdental cleaning tools are needed in the future.

## 5. Conclusions

With the limitations of the study, we conclude that OIs as adjuncts to toothbrushing are significantly more effective than toothbrushing alone in controlling plaque and improving gingival health. Its efficacy was positively correlated with irrigation pressure and the amount of plaque. Furthermore, the application of OIs was safe during the 12 weeks observation period, and with its good compliance, OIs could be effective adjunct cleaning tools.

## Figures and Tables

**Table 1:** Demographic characteristics and the periodontal indices at baseline for two groups (full analysis set).

Parameter		Test (n = 45)	Control (n = 43)	*p*
Gender, n (%)	Male	17 (37.8%)	15 (34.9%)	0.827 ^a^
	Female	28 (62.2%)	28 (65.1%)	
Ethnicity, n (%)	Han ethnic group	44 (97.8%)	40 (93.0%)	0.355 ^b^
	Minority ethnic group	1 (2.2%)	3 (7.0%)	
Education level, n (%)	Elementary school and below	0 (0.0%)	1 (2.3%)	0.475 ^b^
	Junior high school	0 (0.0%)	1 (2.3%)	
	Senior High School	2 (4.4%)	0 (0.0%)	
	College	4 (8.9%)	2 (4.7%)	
	Bachelor	20 (44.4%)	17 (39.5%)	
	Master and above	19 (42.2%)	22 (51.2%)	
Age (Mean ± SD)		30.67 ± 10.05	30.49 ± 11.64	0.314 ^c^
T-QH (Mean ± SD)		2.57 ± 0.55	2.62 ± 0.51	0.684 ^d^
MGI (Mean ± SD)		2.07 ± 0.34	2.14 ± 0.31	0.385 ^d^
BI (Mean ± SD)		1.80 ± 0.41	1.83 ± 0.45	0.907 ^c^
BOP% (Mean ± SD)		42.05 ± 15.45	43.01 ± 17.97	0.789 ^d^

^a^ Chi-squared test. ^b^ Fisher’s exact test. ^c^ Wilcoxon rank-sum test. ^d^ Independent samples *t* test. Abbreviations: T-QH, Turesky-Modified Quigley-Hein Plaque Index; MGI, Modified Gingival Index; BI, Bleeding Index; BOP%, percentage of sites with bleeding on probing.

**Table 2:** The periodontal indices for two groups at follow-up (full analysis set).

Index	Time Point	Group	Mean ± SD	Mean Efficacy *	*p*
T−QH	4 weeks	test	2.18 ± 0.42	−6.84%	0.102 ^a^
		control	2.34 ± 0.49		
	8 weeks	test	2.20 ± 0.49	−9.09%	**0.033 ^a^**
		control	2.42 ± 0.47		
	12 weeks	test	2.27 ± 0.52	−10.98%	**0.006 ^a^**
		control	2.55 ± 0.43		
MGI	4 weeks	test	1.83 ± 0.27	−7.58%	**0.017 ^a^**
		control	1.98 ± 0.31		
	8 weeks	test	1.71 ± 0.28	−11.40%	**<0.001 ^a^**
		control	1.93 ± 0.28		
	12 weeks	test	1.68 ± 0.28	−12.50%	**<0.001 ^a^**
		control	1.92 ± 0.26		
BI	4 weeks	test	1.30 ± 0.24	−17.72%	**0.001 ^b^**
		control	1.58 ± 0.40		
	8 weeks	test	1.20 ± 0.23	−18.92%	**<0.001 ^b^**
		control	1.48 ± 0.33		
	12 weeks	test	1.20 ± 0.26	−20.00%	**<0.001 ^b^**
		control	1.50 ± 0.33		
BOP%	4 weeks	test	19.53 ± 11.96	−43.52%	**0.001 ^b^**
		control	34.58 ± 21.05		
	8 weeks	test	14.14 ± 12.35	−56.56%	**<0.001 ^b^**
		control	32.55 ± 21.97		
	12 weeks	test	13.89 ± 13.46	−58.35%	**<0.001 ^b^**
		control	33.35 ± 21.89		

^a^ Independent samples *t* test. ^b^ Wilcoxon rank-sum test. * Mean efficacy = (test − control)/control × 100%. Abbreviations: T-QH, Turesky-Modified Quigley-Hein Plaque Index; MGI, Modified Gingival Index; BI, Bleeding Index; BOP%, percentage of sites with bleeding on probing. Bold denotes statistical significance at *p* < 0.05.

**Table 3:** The reduction in percentage of the periodontal indices for two groups at follow-up (full analysis set).

Index	Time Point	Group	Mean ± SD	Mean Difference *	*p*
T-QH	Baseline–	test	9.96 ± 19.46	9.85	**0.021 ^a^**
	12 weeks	control	0.11 ± 19.91		
	Baseline–	test	13.41 ± 16.98	3.94	0.275 ^a^
	4 weeks	control	9.47 ± 16.61		
	4 weeks–	test	−1.06 ± 11.44	3.68	0.214 ^b^
	8 weeks	control	−4.74 ± 12.84		
	8 weeks–	test	−3.27 ± 9.27	3.41	0.498 ^b^
	12 weeks	control	−6.68 ± 15.44		
MGI	Baseline–	test	18.13 ± 11.53	8.96	**<0.001 ^a^**
	12 weeks	control	9.17 ± 11.36		
	Baseline–	test	10.91 ± 10.27	4.46	0.065 ^a^
	4 weeks	control	6.45 ± 12.03		
	4 weeks–	test	6.62 ± 9.90	4.60	**0.007 ^b^**
	8 weeks	control	2.02 ± 8.22		
	8 weeks–	test	1.17 ± 7.10	0.96	0.541 ^a^
	12 weeks	control	0.21 ± 7.62		
BI	Baseline–	test	32.47 ± 8.39	15.83	**<0.001 ^a^**
	12 weeks	control	16.64 ± 13.44		
	Baseline–	test	26.74 ± 7.95	14.08	**<0.001 ^b^**
	4 weeks	control	12.66 ± 14.08		
	4 weeks–	test	7.26 ± 10.27	2.40	0.115 ^b^
	8 weeks	control	4.86 ± 10.87		
	8 weeks–	test	−0.50 ± 12.44	0.72	0.391 ^b^
	12 weeks	control	−1.22 ± 8.49		
BOP%	Baseline–	test	70.97 ± 20.64	45.80	**<0.001 ^b^**
	12 weeks	control	25.17 ± 40.38		
	Baseline–	test	55.79 ± 18.31	33.19	**<0.001 ^a^**
	4 weeks	control	22.60 ± 36.03		
	4 weeks–	test	31.54 ± 37.71	26.42	**<0.001 ^a^**
	8 weeks	control	5.12 ± 32.22		
	8 weeks–	test	−4.92 ± 74.52	3.59	0.105 ^b^
	12 weeks	control	−8.51 ± 45.60		

^a^ Independent samples *t* test. ^b^ Wilcoxon rank-sum test. * Mean difference = test − control. Abbreviations: T-QH, Turesky-Modified Quigley-Hein Plaque Index; MGI, Modified Gingival Index; BI, Bleeding Index; BOP%, percentage of sites with bleeding on probing. Bold denotes statistical significance at *p* < 0.05.

**Table 4:** Comparison of adverse events between for two groups (safety set).

		Test	Control	Overall	*p* ^a^
Adverse events	no	27 (60.0%)	24 (55.8%)	51	0.829
	yes	18 (40.0%)	19 (44.2%)	37	
	Overall	45	43	88	

^a^ Chi-squared test.

**Table 5:** Pain and dentin hypersensitivity symptoms of participants in two groups (safety set).

	Time point	Group	Mean ± SD	Q_1_	Q_2_	Q_3_	*p* ^a^
Pain	Baseline	test	1.49 ± 8.13	0	0	0	0.412
		control	1.80 ± 6.28	0	0	0	
	4 weeks	test	1.36 ± 8.79	0	0	0	0.982
		control	1.84 ± 12.20	0	0	0	
	8 weeks	test	0.52 ± 3.47	0	0	0	0.577
		control	0.20 ± 1.24	0	0	0	
	12 weeks	test	2.42 ± 5.66	0	0	2	0.175
		control	1.90 ± 5.71	0	0	0	
Dentin hypersensitivity	Baseline	test	10.89 ± 17.69	0	0	25.5	0.905
		control	11.22 ± 19.22	0	0	22	
	4 weeks	test	4.91 ± 12.94	0	0	0	0.403
		control	8.36 ± 19.90	0	0	2	
	8 weeks	test	6.20 ± 11.67	0	0	8.25	0.070
		control	2.59 ± 8.35	0	0	0	
	12 weeks	test	9.11 ± 14.59	0	0	11.25	0.716
		control	9.90 ±18.34	0	0	14.25	

^a^ Wilcoxon rank-sum test. Abbreviations: Q_1_, 25th percentile; Q_2_, median; Q_3_, 75th percentile.

**Table 6:** The compliance of participants in two groups (full analysis set).

	Group	Mean ± SD	Q_1_	Q_2_	Q_3_	*p* ^a^
Toothbrushing	test	98.91 ± 1.78	98.80	100.00	100.00	0.355
	control	99.33 ± 1.08	98.81	100.00	100.00	
Oral irrigation	test	95.90 ± 9.14	96.45	98.81	100.00	

^a^ Wilcoxon rank-sum test. Abbreviations: Q_1_, 25th percentile; Q_2_, median; Q_3_, 75th percentile.

## Data Availability

Data for this study can be obtained by contacting the corresponding author under reasonably requested.

## Supplementary Materials

The following supporting information can be downloaded at: https://www.mdpi.com/article/10.3390/ijerph20043726/s1, Table S1: The periodontal indices for two groups at follow-up (per-protocol set); Table S2: The reduction in percentage of the periodontal indices for two groups at follow-up (per-protocol set); Figure S1: The correlation between the reduction in percentage at the baseline-12 weeks period and the average irrigation pressures (full analysis set); Figure S2: The correlation between the reduction in percentage at the baseline-12 weeks period and the average irrigation pressures (per-protocol set); Figure S3: The correlation between the reduction in percentage at the baseline-12 weeks period and the severity at baseline (per-protocol set); Figure S4: Scatter diagram of periodontal indices for two groups at follow-up (per-protocol set).

## References

[1] Ng E., Tay J.R.H., Ong M.M.A. (2021). Minimally Invasive Periodontology: A Treatment Philosophy and Suggested Approach. Int. J. Dent..

[2] Jiao J., Jing W., Si Y., Feng X., Tai B., Hu D., Lin H., Wang B., Wang C., Zheng S. (2021). The prevalence and severity of periodontal disease in Mainland China: Data from the Fourth National Oral Health Survey (2015–2016). J. Clin. Periodontol..

[3] Kinane D.F., Stathopoulou P.G., Papapanou P.N. (2017). Periodontal diseases. Nat. Rev. Dis. Prim..

[4] Ng E., Tay J.R.H., Balan P., Ong M.M.A., Bostanci N., Belibasakis G.N., Seneviratne C.J. (2021). Metagenomic sequencing provides new insights into the subgingival bacteriome and aetiopathology of periodontitis. J. Periodontal Res..

[5] Van der Weijden F.A., Slot D.E. (2015). Efficacy of homecare regimens for mechanical plaque removal in managing gingivitis a meta review. J. Clin. Periodontol..

[6] Sälzer S., Graetz C., Dörfer C.E., Slot D.E., Van der Weijden F.A. (2020). Contemporary practices for mechanical oral hygiene to prevent periodontal disease. J. Clin. Periodontol..

[7] Gennai S., Nisi M., Perić M., Marhl U., Izzetti R., Tonelli M., Petrini M., Graziani F. (2022). Interdental plaque reduction after the use of different devices in patients with periodontitis and interdental recession: A randomized clinical trial. Int. J. Dent. Hyg..

[8] Ng E., Lim L.P. (2019). An Overview of Different Interdental Cleaning Aids and Their Effectiveness. Dent. J..

[9] Sälzer S., Slot D.E., Van der Weijden F.A., Dörfer C.E. (2015). Efficacy of inter-dental mechanical plaque control in managing gingivitis—A meta-review. J. Clin. Periodontol..

[10] Zhao Q., Wang S.B., Xu G., Song Y., Han X., Liu Z., Zhou X., Zhang T., Huang K., Yang T. (2019). Periodontal health: A national cross-sectional study of knowledge, attitudes and practices for the public oral health strategy in China. J. Clin. Periodontol..

[11] Greenstein G. (2005). Position paper: The role of supra- and subgingival irrigation in the treatment of periodontal diseases. J. Periodontol..

[12] Worthington H.V., MacDonald L., Poklepovic Pericic T., Sambunjak D., Johnson T.M., Imai P., Clarkson J.E. (2019). Home use of interdental cleaning devices, in addition to toothbrushing, for preventing and controlling periodontal diseases and dental caries. Cochrane Database Syst. Rev..

[13] Husseini A., Slot D.E., Van der Weijden G.A. (2008). The efficacy of oral irrigation in addition to a toothbrush on plaque and the clinical parameters of periodontal inflammation: A systematic review. Int. J. Dent. Hyg..

[14] Walsh M., Heckman B., Leggott P., Armitage G., Robertson P.B. (1989). Comparison of manual and power toothbrushing, with and without adjunctive oral irrigation, for controlling plaque and gingivitis. J. Clin. Periodontol..

[15] Newman M.G., Flemmig T.F., Nachnani S., Rodrigues A., Calsina G., Lee Y.S., de Camargo P., Doherty F.M., Bakdash M.B. (1990). Irrigation with 0.06% chlorhexidine in naturally occurring gingivitis. II. 6 months microbiological observations. J. Periodontol..

[16] Flemmig T.F., Newman M.G., Doherty F.M., Grossman E., Meckel A.H., Bakdash M.B. (1990). Supragingival irrigation with 0.06% chlorhexidine in naturally occurring gingivitis. I. 6 month clinical observations. J. Periodontol..

[17] Jolkovsky D.L., Waki M.Y., Newman M.G., Otomo-Corgel J., Madison M., Flemmig T.F., Nachnani S., Nowzari H. (1990). Clinical and microbiological effects of subgingival and gingival marginal irrigation with chlorhexidine gluconate. J. Periodontol..

[18] Hugoson A. (1978). Effect of the Water Pik device on plaque accumulation and development of gingivitis. J. Clin. Periodontol..

[19] Newman M.G., Cattabriga M., Etienne D., Flemmig T., Sanz M., Kornman K.S., Doherty F., Moore D.J., Ross C. (1994). Effectiveness of adjunctive irrigation in early periodontitis: Multi-center evaluation. J. Periodontol..

[20] Walsh T.F., Glenwright H.D., Hull P.S. (1992). Clinical effects of pulsed oral irrigation with 0.2% chlorhexidine digluconate in patients with adult periodontitis. J. Clin. Periodontol..

[21] Goyal C.R., Qaqish J.G., Schuller R., Lyle D.M. (2018). Evaluation of the Addition of a Water Flosser to Manual Brushing on Gingival Health. J. Clin. Dent..

[22] Lyle D.M., Qaqish J.G., Goyal C.R., Schuller R. (2020). Efficacy of the Use of a Water Flosser in Addition to an Electric Toothbrush on Clinical Signs of Inflammation: 4-Week Randomized Controlled Trial. Compend. Contin. Educ. Dent..

[23] Goyal C.R., Lyle D.M., Qaqish J.G., Schuller R. (2016). Comparison of Water Flosser and Interdental Brush on Reduction of Gingival Bleeding and Plaque: A Randomized Controlled Pilot Study. J. Clin. Dent..

[24] Meklas J.F., Stewart J.L. (1972). Investigation of the safety and effectiveness of an oral irrigating device. J. Periodontol..

[25] Rmaile A., Carugo D., Capretto L., Aspiras M., De Jager M., Ward M., Stoodley P. (2014). Removal of interproximal dental biofilms by high-velocity water microdrops. J. Dent. Res..

[26] Sharma N.C., Lyle D.M., Qaqish J.G., Schuller R. (2012). Comparison of two power interdental cleaning devices on the reduction of gingivitis. J. Clin. Dent..

[27] Lobene R.R., Weatherford T., Ross N.M., Lamm R.A., Menaker L. (1986). A modified gingival index for use in clinical trials. Clin. Prev. Dent..

[28] Mazza J.E., Newman M.G., Sims T.N. (1981). Clinical and antimicrobial effect of stannous fluoride on periodontitis. J. Clin. Periodontol..

[29] Turesky S., Gilmore N.D., Glickman I. (1970). Reduced plaque formation by the chloromethyl analogue of victamine C. J. Periodontol..

[30] Reips U.D., Funke F. (2008). Interval-level measurement with visual analogue scales in Internet-based research: VAS Generator. Behav. Res. Methods.

[31] Mostazir M., Taylor G., Henley W.E., Watkins E.R., Taylor R.S. (2021). Per-Protocol analyses produced larger treatment effect sizes than intention to treat: A meta-epidemiological study. J. Clin. Epidemiol..

[32] Cutler C.W., Stanford T.W., Abraham C., Cederberg R.A., Boardman T.J., Ross C. (2000). Clinical benefits of oral irrigation for periodontitis are related to reduction of pro-inflammatory cytokine levels and plaque. J. Clin. Periodontol..

[33] Al-Mubarak S., Ciancio S., Aljada A., Mohanty P., Ross C., Dandona P. (2002). Comparative evaluation of adjunctive oral irrigation in diabetics. J. Clin. Periodontol..

[34] Tecco S., Nota A., D’Amicantonio T., Pittari L., Monti M., Polizzi E. (2022). Effects of an Ozonated Water Irrigator on the Plaque Index and Bleeding Index of Pregnant Women. J. Clin. Med..

[35] De David S.C., Mário T.G., De Freitas G.C., Kantorski K.Z., Wikesjö U.M.E., Moreira C.H.C. (2018). Correlation between plaque control and gingival health using short and extended oral hygiene intervals. Clin. Oral Investig..

[36] Sedgwick P., Greenwood N. (2015). Understanding the Hawthorne effect. BMJ.

[37] Gillette W.B., Van House R.L. (1980). Ill effects of improper oral hygeine procedure. J. Am. Dent. Assoc..

[38] Bhaskar S.N., Cutright D.E., Gross A., Frisch J., Beasley J.D., Perez B. (1971). Water jet devices in dental practice. J. Periodontol..

[39] Boyd R.L., Leggott P., Quinn R., Buchanan S., Eakle W., Chambers D. (1985). Effect of self-administered daily irrigation with 0.02% SnF2 on periodontal disease activity. J. Clin. Periodontol..

[40] Cobb C.M., Rodgers R.L., Killoy W.J. (1988). Ultrastructural examination of human periodontal pockets following the use of an oral irrigation device in vivo. J. Periodontol..

[41] Teixeira D.N.R., Zeola L.F., Machado A.C., Gomes R.R., Souza P.G., Mendes D.C., Soares P.V. (2018). Relationship between noncarious cervical lesions, cervical dentin hypersensitivity, gingival recession, and associated risk factors: A cross-sectional study. J. Dent..

[42] Flemmig T.F., Epp B., Funkenhauser Z., Newman M.G., Kornman K.S., Haubitz I., Klaiber B. (1995). Adjunctive supragingival irrigation with acetylsalicylic acid in periodontal supportive therapy. J. Clin. Periodontol..

[43] Trombelli L., Farina R., Silva C.O., Tatakis D.N. (2018). Plaque-induced gingivitis: Case definition and diagnostic considerations. J. Clin. Periodontol..

[44] Stone A.A., Shiffman S., Schwartz J.E., Broderick J.E., Hufford M.R. (2002). Patient non-compliance with paper diaries. BMJ.

[45] Daniëls N.E.M., Hochstenbach L.M.J., van Zelst C., van Bokhoven M.A., Delespaul P., Beurskens A. (2021). Factors That Influence the Use of Electronic Diaries in Health Care: Scoping Review. JMIR Mhealth Uhealth.

[46] Fabbri S., Johnston D.A., Rmaile A., Gottenbos B., De Jager M., Aspiras M., Starke E.M., Ward M.T., Stoodley P. (2016). High-Velocity Microsprays Enhance Antimicrobial Activity in Streptococcus mutans Biofilms. J. Dent. Res..

[47] Chaves E.S., Kornman K.S., Manwell M.A., Jones A.A., Newbold D.A., Wood R.C. (1994). Mechanism of irrigation effects on gingivitis. J. Periodontol..

[48] Fine J.B., Harper D.S., Gordon J.M., Hovliaras C.A., Charles C.H. (1994). Short-term microbiological and clinical effects of subgingival irrigation with an antimicrobial mouthrinse. J. Periodontol..

[49] Ramseier C.A., Petitat C., Trepp S., Lang N.P., Eick S., Adam R., Ccahuana-Vasquez R.A., Barker M.L., Timm H., Klukowska M. (2021). Clinical Parameters and Oral Fluid Biomarkers in Gingivitis Subjects using an Electric Toothbrush with Irrigator vs a Manual Toothbrush Alone over 8 Weeks: A Randomised Controlled Clinical Trial. Oral Health Prev. Dent..

