# Supplementary material for: The Efficacy and Safety of Oral Irrigator on the Control of Dental Plaque and Gingivitis: A Randomized, Single-Blind, Parallel-Group Clinical Trial
Source: Int J Environ Res Public Health. 2023 Feb 20;20(4):3726. doi: 10.3390/ijerph20043726 (PMC9965011; doi:10.3390/ijerph20043726)
Supplement: Supplementary file 1 [file ijerph-20-03726-s001.zip › ijerph-2176762-supplementary.pdf]

Supplementary Materials

The efficacy and safety of oral irrigator on the control of dental plaque and gingivitis: a randomized, single-blind, parallel-group clinical trial

Xiaolin Ren [1,†], Jing He [1,†], Ran Cheng [1], Yulun Chen [1], Yong Xiang [1], Yuhan Zhang [1], Sulan Jiang [1], Jia Li[2], Li Cheng [1,*], Tao Hu[1]

[1] State Key Laboratory of Oral Diseases, National Clinical Research Center for Oral Diseases, Department of Preventive Dentistry, West China Hospital of Stomatology, Sichuan University, Chengdu, 610041 China.

[2] State Institute of Drug Clinical Trial, West China Hospital of Stomatology, Sichuan University, Chengdu, 610041 China.

[*] Correspondence: dentistcl@scu.edu.cn

[†] These authors contributed equally to this work.

Table S1 The periodontal indices for two groups at follow-up (per-protocol set)

Index	Time point	Group	Mean ± SD	Mean Efficacy*	*P*
T-QH	4 weeks	test	2.18 ± 0.45	-5.63%	0.266[a]
		control	2.31 ± 0.50		
	8 weeks	test	2.18 ± 0.52	-9.92%	**0.042**[a]
		control	2.42 ± 0.47		
	12 weeks	test	2.25 ± 0.54	-12.45%	**0.006**[a]
		control	2.57 ± 0.42		
MGI	4 weeks	test	1.82 ± 0.30	-6.67%	0.082[a]
		control	1.95 ± 0.30		
	8 weeks	test	1.70 ± 0.29	-10.99%	**0.003**[a]
		control	1.91 ± 0.28		
	12 weeks	test	1.67 ± 0.27	-11.64%	**< 0.001**[a]
		control	1.89 ± 0.26		
BI	4 weeks	test	1.31 ± 0.24	-13.25%	**0.014**[b]
		control	1.51 ± 0.35		
	8 weeks	test	1.20 ± 0.22	-17.24%	**< 0.001**[b]
		control	1.45 ± 0.32		
	12 weeks	test	1.20 ± 0.27	-17.81%	**< 0.001**[b]
		control	1.46 ± 0.33		
BOP%	4 weeks	test	20.40 ± 12.30	-37.96%	**0.020**[b]
		control	32.88 ± 21.33		
	8 weeks	test	14.54 ± 12.24	-54.15%	**< 0.001**[b]
		control	31.71 ± 22.75		
	12 weeks	test	14.12 ± 13.67	-56.71%	**< 0.001**[b]
		control	32.62 ± 22.69		

[a] Independent samples *t* test

[b] Wilcoxon rank-sum test

*Mean efficacy = (test − control)/control × 100%

Abbreviations: T-QH, Turesky-Modified Quigley-Hein Plaque Index; MGI, Modified Gingival Index; BI, Bleeding Index; BOP%, percentage of sites with bleeding on probing. Bold denotes statistical significance at $P < 0.05$.

Table S2 The reduction in percentage of the periodontal indices for two groups at follow-up (per-protocol set)

Index	Time point	Group	Mean ± SD	Mean Difference*	*P*
T-QH	Baseline-	test	10.00 ± 21.93	12.28	**0.014**[a]
	12 weeks	control	-2.28 ± 19.35		
	Baseline-	test	12.62 ± 19.02	3.87	0.372[a]
	4 weeks	control	8.75 ±17.32		
	4 weeks-	test	0.46 ±10.20	6.42	**0.040**[b]
	8 weeks	control	-5.96 ±12.82		
	8 weeks-	test	-3.57 ±9.86	3.98	0.541[b]
	12 weeks	control	-7.55 ±16.24		
MGI	Baseline-	test	20.73 ±11.24	11.24	**< 0.001**[a]
	12 weeks	control	9.49 ±11.75		
	Baseline-	test	13.65 ±8.98	6.44	**0.014**[a]
	4 weeks	control	7.21 ±12.14		
	4 weeks-	test	6.61 ±8.86	5.10	**0.008**[b]
	8 weeks	control	1.51 ±8.57		
	8 weeks-	test	1.48 ±7.21	1.24	0.501[a]
	12 weeks	control	0.24 ±8.12		
BI	Baseline-	test	33.72 ±8.00	17.95	**< 0.001**[a]
	12 weeks	control	15.77 ±11.41		
	Baseline-	test	27.40 ±7.80	14.02	**< 0.001**[a]
	4 weeks	control	13.38 ±10.51		
	4 weeks-	test	8.12 ±8.37	4.65	**0.033**[a]
	8 weeks	control	3.47 ±9.48		
	8 weeks-	test	-0.44 ±13.93	0.94	0.387[b]
	12 weeks	control	-1.38 ±9.03		
BOP%	Baseline-	test	71.43 ±19.84	46.94	**< 0.001**[b]
	12 weeks	control	24.49 ±40.79		
	Baseline-	test	54.98 ±17.58	31.34	**< 0.001**[a]
	4 weeks	control	23.64 ±35.40		
	4 weeks-	test	34.22 ±33.66	30.62	**< 0.001**[a]
	8 weeks	control	3.60 ±32.91		
	8 weeks-	test	-1.08 ±61.15	8.55	0.181[b]
	12 weeks	control	-9.63 ±48.47		

[a] Independent samples *t* test

[b] Wilcoxon rank-sum test

* Mean difference = test – control

Abbreviations: T-QH, Turesky-Modified Quigley-Hein Plaque Index; MGI, Modified Gingival Index; BI, Bleeding Index; BOP%, percentage of sites with bleeding on probing. Bold denotes statistical significance at $P < 0.05$.

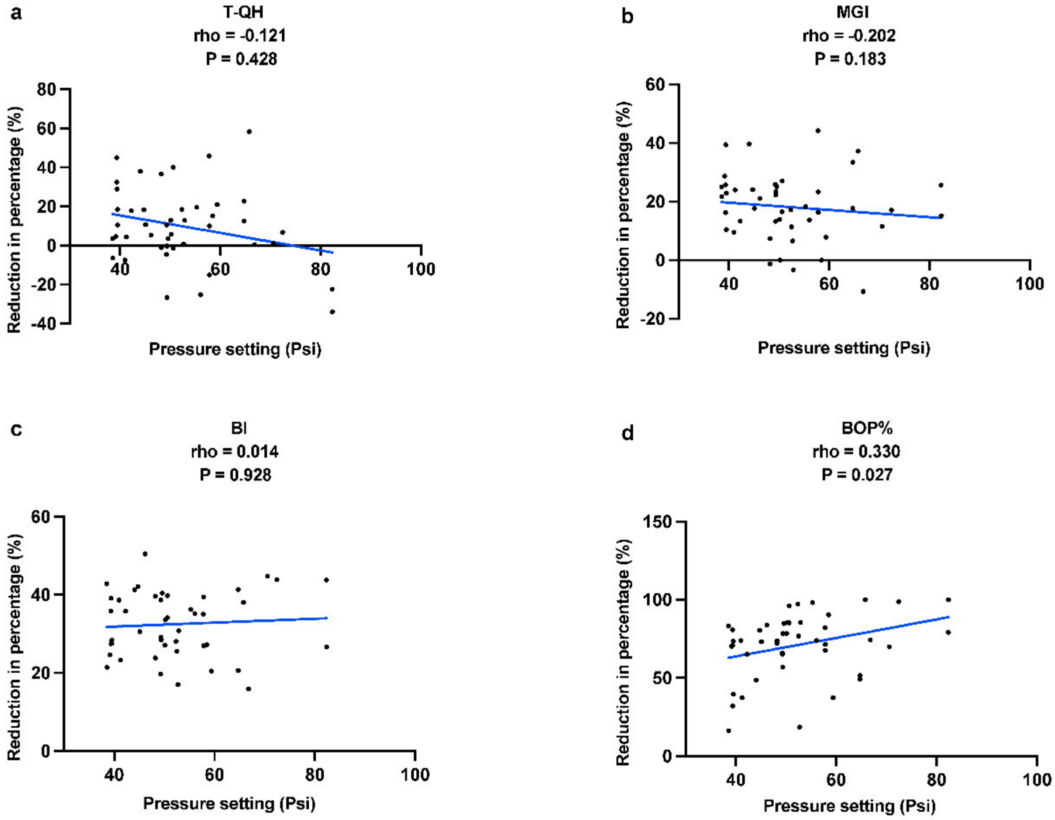

Figure S1 The correlation between the reduction in percentage at the baseline-12 weeks period and the average irrigation pressures (full analysis set). (a) T-QH, Turesky-Modified Quigley-Hein Plaque Index; (b) MGI, Modified Gingival Index; (c) BI, Bleeding Index; (d) BOP%, percentage of sites with bleeding on probing. rho, Spearman correlation coefficient. Psi, Pound per square inch.

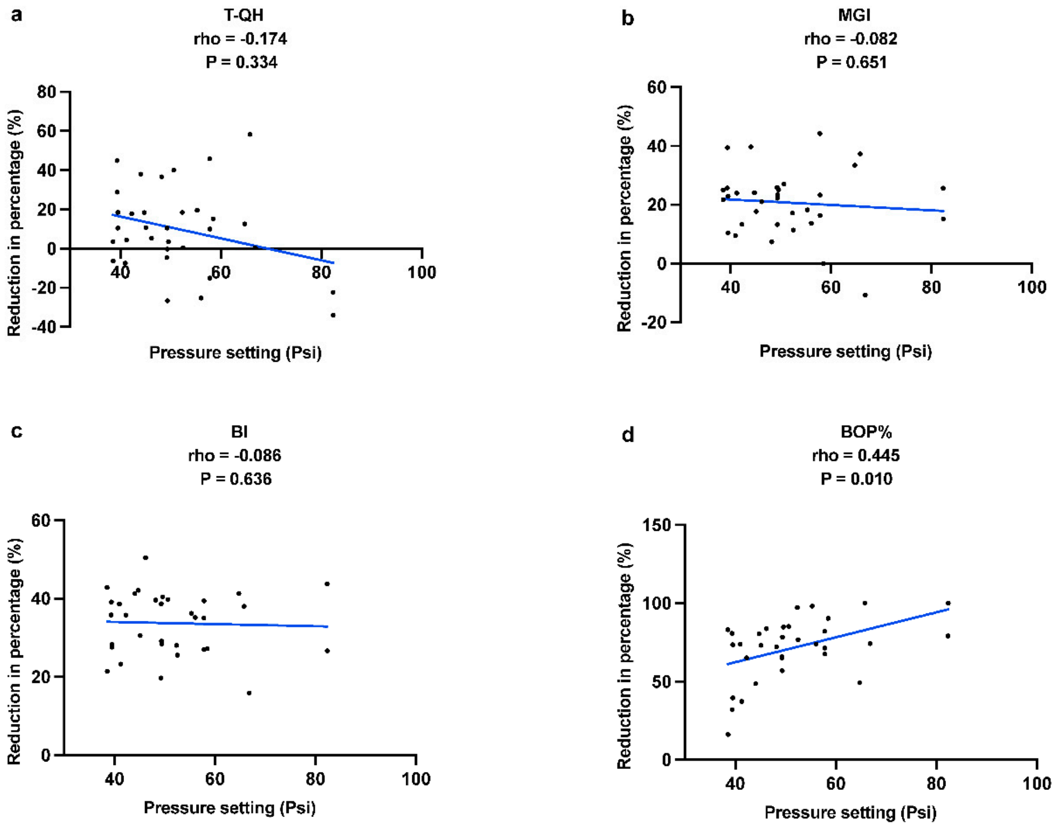

Figure S2 The correlation between the reduction in percentage at the baseline-12 weeks period and the average irrigation pressures (per-protocol set). (a) T-QH, Turesky-Modified Quigley-Hein Plaque Index; (b) MGI, Modified Gingival Index; (c) BI, Bleeding Index; (d) BOP%, percentage of sites with bleeding on probing. rho, Spearman correlation coefficient. Psi, Pound per square inch.

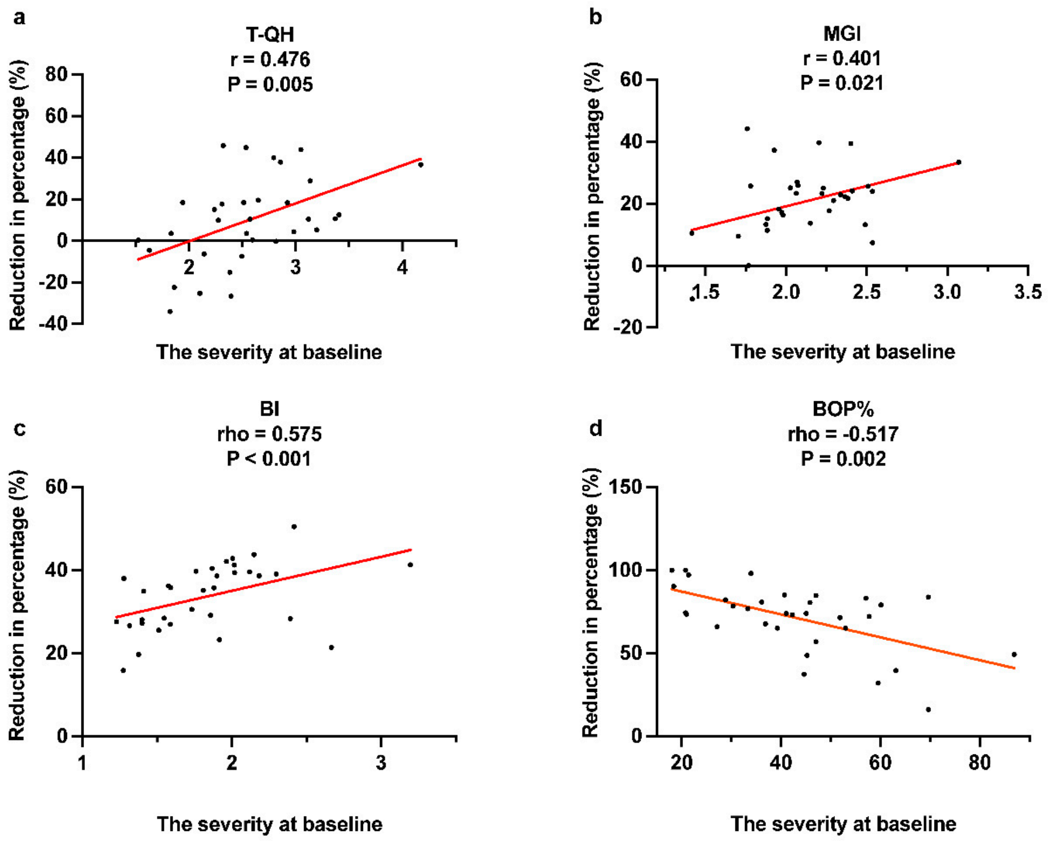

Figure S3 The correlation between the reduction in percentage at the baseline-12 weeks period and the severity at baseline (per-protocol set). (a) T-QH, Turesky-Modified Quigley-Hein Plaque Index; (b) MGI, Modified Gingival Index; (c) BI, Bleeding Index; (d) BOP%, percentage of sites with bleeding on probing. Pearson correlation analysis was adopted in (a,b). Spearman correlation analysis was adopted in (c,d). r, Pearson correlation coefficient; rho, Spearman correlation coefficient.

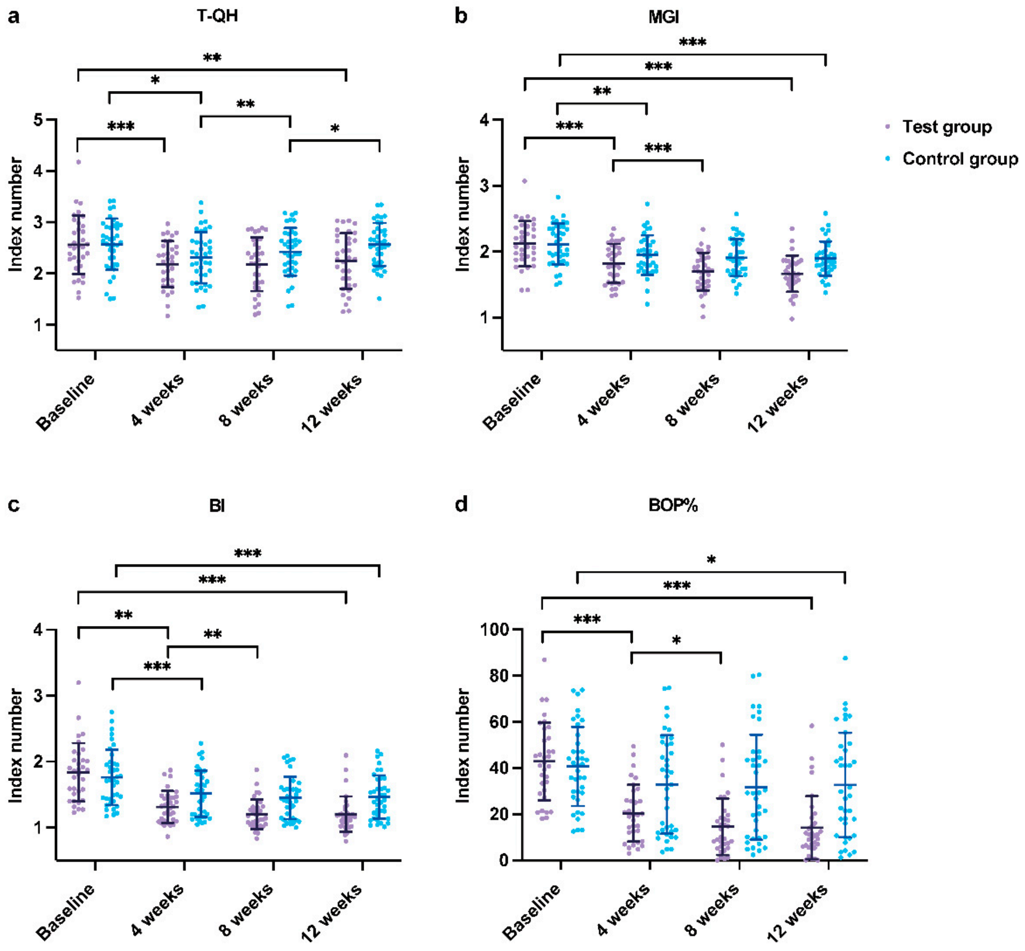

Figure S4 Scatter diagram of periodontal indices for two groups at follow-up (per-protocol set). (a) T-QH, Turesky-Modified Quigley-Hein Plaque Index; (b) MGI, Modified Gingival Index; (c) BI, Bleeding Index; (d) BOP%, percentage of sites with bleeding on probing. Intragroup comparisons in (a,b) were performed using Two-way Repeated Measures ANOVA and post hoc tests with Bonferroni correction. Intragroup comparisons in (c,d) were performed using Friedman's test (*$P < 0.05$, **$P < 0.01$, ***$P < 0.001$).
